# Health implications of lower extremity amputations in Jordan: A retrospective analysis of demographic patterns and causes

**DOI:** 10.1371/journal.pone.0329149

**Published:** 2025-07-24

**Authors:** Mahmoud Alfatafta, Nizar Alsubahi, Huda Alfatafta, Huthaifa Atallah, Amneh Alshawabka, Anthony McGarry, Alaeddin Ahmad

**Affiliations:** 1 Department of Prosthetics and Orthotics, School of Rehabilitation Sciences, The University of Jordan, Amman, Jordan; 2 Department of Health Services and Hospitals Administration, Faculty of Economics and Administration, King Abdulaziz University, Jeddah, Saudi Arabia; 3 Department of Health Services Research, Care and Public Health Research Institute—CAPHRI, Faculty of Health, Medicine and Life Sciences, Maastricht University Medical Center, Maastricht University, Maastricht, The Netherlands; 4 Faculty of Health Sciences, University of Pecs, Pecs, Hungary; 5 Biomedical Engineering, The University of Strathclyde, Strathclyde, Glasgow, United Kingdom; 6 Department of Marketing, School of Business, The University of Jordan, Amman, Jordan; Brunel University London, UNITED KINGDOM OF GREAT BRITAIN AND NORTHERN IRELAND

## Abstract

Lower extremity amputation (LEA) is a significant health concern in Jordan, yet comprehensive data on its demographic and clinical characteristics remain limited. This retrospective analysis evaluated 893 LEA cases collected from Al-Basheer Hospital and six private prosthetic clinics in Amman between 2017 and 2023. Transtibial amputations (68.99%) were the most common, followed by transfemoral amputations (24.53%). Males were three times more likely than females to undergo LEA, with an overall mean age of 48.43 years (SD = 20.42). Diabetes mellitus (55.88%) was the leading cause, followed by cancer (18.48%) and trauma (11.65%). Age and cause were significantly associated (p < 0.01); DM-related amputations were more prevalent among older adults (mean age 62.04 years). The findings highlight that TT amputations in older males with diabetes represent the most common LEA profile in Jordan. Targeted public health initiatives including diabetic foot care education, early detection, and regular screenings, are urgently needed to reduce LEA incidence in the country.

## Introduction

Lower extremity amputation (LEA) is an increasingly prevalent health concern in Jordan [1]. Various medical conditions, such as diabetes mellitus (DM), peripheral vascular disease (PVD), and injuries have contributed to the rising incidence of LEA [2]. LEA significantly reduces locomotor functionality, impacting overall physical abilities [3]. Restoring function, reaching a satisfactory functional level, and improving participation are the primary goals of prosthetic fitting [4].

The global incidence of LEA is expected to rise significantly by 2050, attributed to two key factors: the aging population and the increased prevalence of DM worldwide [5]. The most current comprehensive study about the demographic distribution in Jordan was conducted 14 years ago, highlighting a significant gap.

As the demand for prosthetic services continues to rise, understanding the characteristics of the population relying on these devices becomes crucial for improving rehabilitation outcomes. Many articles document the incidence of lower extremity amputations in Jordan. A retrospective study across 371 recorded cases that conducted by [6] between 1996 and 1998 across seventeen health centers in Amman concluded that male to female ratio was 2.7:1, with a mean age of 59.6 years, while the incidence rate of LEA was 23.6/100.000/year [6]. Another study that was conducted in southern Jordan from 1998 to 2006 showed that the mean age of individual at time of amputation was 46 years [7], with a higher prevalence of LEA in males than females. Furthermore, DM and trauma were reported to be the main causes of amputations [7]. A study carried out in Amman over seven years and six months among 687 people with amputation revealed that trauma was the most prevalent cause of amputation (47.3%), followed by DM (33.3%), infections, malignancies, congenital disorders, and PVD (19.4%) [8]. Another study conducted in the northern part of Jordan among 235 people with amputation found that trauma was the predominant contributor to LEA (51%) then DM (32.3%), and infections (16.7%) [8]. All articles described transtibial amputation as the primary level of LEA [9].

Jordan and majority of the other developing countries have increased incidence of major LEA due to diabetes mellitus. The lack of early-stage assessment and delayed referral reported to play a role in this issue. In contrast, in developed countries like USA and UK, major LEA rate decreased with a moderate increase in minor LEA from 2010 until 2020 [10]. This may be due to the preventive strategies emerged such as programs like the National Diabetes Prevention Program (NDPP) in the USA which focus on early detection and patient education, aiming to reduce diabetes-related amputations [11]. Similarly, the UK’s National Health Service (NHS) employs diabetic foot screening as part of a broader initiative to prevent LEAs, achieving a 25% reduction in major amputations over a decade [10].

This study aims to determine the predominant anatomical levels of lower extremity amputations (LEAs), analyze demographic distributions by age and gender, identify key etiological factors, and assess the relationships among these variables to provide a comprehensive understanding of LEA patterns in Jordan.

## Methods

### Data source

This retrospective cross-sectional study was conducted in Amman, Jordan, and included patients who received prosthetic limb services at six private prosthetic clinics and Al-Basheer Hospital, the largest public provider of such services in the country. The dataset spanned seven years, from January 1, 2017, to December 31, 2023.

All available patient records were reviewed. Records were included if they had complete data on four key variables: age, gender, cause of amputation, and level of amputation. Records missing one or more of these variables were excluded from analysis.

The clinics and hospital included in this study represent the primary providers of prosthetic services in Jordan, covering both public and private sectors. This broad coverage ensures that the sample is reflective of the diversity of the Jordanian population in terms of socioeconomic background, geographic distribution, and access to healthcare. As such, the findings can reasonably be extrapolated to represent the wider demographic trends of prosthetic limb users in Jordan.

The data were anonymized prior to analysis, no identifiable information was accessible to the researchers. As such, individual informed consent for publication was not required. However, the use of de-identified data for academic purposes aligns with institutional policies and ethical standards. The hospitals and clinics providing the data had existing agreements allowing for the academic and research use of such records in compliance with their ethical and data-sharing protocols. The data were accessed during the period between 30/11/2023 until 27/11/2024.

A formal test for missing data mechanisms (e.g., MCAR or MAR) was not conducted due to the retrospective nature of the dataset and the lack of auxiliary variables necessary to support such analysis. Based on a preliminary review of the source files and input from data providers, the pattern of missingness appeared to be random and primarily administrative in nature such as incomplete file transfers or issues with handwritten documentation. This suggests a limited risk of systematic bias. The implications of this assumption are further discussed in the study’s limitations section.

This study was conducted and reported in accordance with the Strengthening the Reporting of Observational Studies in Epidemiology (STROBE) guidelines for cross-sectional studies. A completed STROBE checklist is provided in the supporting information.

Ethical approval for this study was obtained from the Institutional Review Board (IRB 2344/2023/47) at the University of Jordan and the Ministry of Health (No. 18624), ensuring compliance with ethical guidelines and the protection of participant rights and welfare. Due to the use of de-identified retrospective data, the requirement for individual informed consent was waived by the Institutional Review Board.

### Data analysis

The data were systematically categorized and transformed into percentages to facilitate comparative analysis. Causes of amputation were classified into Diabetes Mellitus (DM), trauma, cancer, Peripheral Vascular Disease (PVD), and congenital conditions. Relative frequencies for each cause were calculated and expressed as percentages for easy comparison.

To evaluate the relationships among the variables—cause of amputation, level of amputation, age group, gender, and type of amputation—a series of statistical analyses were conducted using IBM SPSS Statistics (Version 27). Initially, the normality of the data distribution was assessed using the Shapiro-Wilk test, which confirmed that the data met the assumption of normality (p > 0.05).

Subsequently, an Analysis of Variance (ANOVA) was performed to examine the associations between the variables. All statistical tests were conducted as two-sided tests, as this is the standard approach unless there is a strong theoretical or clinical justification for using one-sided tests. A significance level of α = 0.05 was adopted for all analyses to determine statistical significance.

Amputation levels were organized into two classification criteria. The first criterion classified the levels of amputation into transtibial (TT) or Below Knee; transfemoral (TF) or above knee, Hip Disarticulation (HD), Knee Disarticulation (KD), and foot amputations, which include ankle disarticulation (Symes); mid-tarsal (Chopart); tarso-metatarsal (Lisfranc), and toe amputations. Amputations were further categorized as major (TT, TF, HD, KD) or minor (foot amputations), providing insights into the severity of limb loss.

Age distribution among prosthesis users was segmented into the following categories to reveal demographic trends: children (0–16 years), young adults (17–30 years), middle-aged adults (31–45 years), and older adults (over 45 years) [12].

## Results

The initial study population comprised 1,069 patients. However, during data cleaning, 176 records were excluded due to missing key variables. The number of missing cases by variable was as follows: age (58 records), gender (51 records), cause of amputation (44 records), and amputation level (32 records). After exclusions (some records missing multiple variables), the final dataset included 893 complete cases. S1 Table (supplementary information) provides a breakdown of the number of missing values for each variable.

The analysis revealed several key demographic and clinical trends in lower extremity amputations (LEA), including variations in amputation level, age, gender, and cause of amputation. Table 1 represents the demographic and clinical characteristics of the patients.

**Table 1:** Demographic and clinical characteristics of amputee patients (N = 893). This table presents the distribution of amputation levels, causes, age groups, and gender in the study population.

Category	Subcategory	Number of Patients (n)	Percentage (%)
Amputation Levels	Transtibial (TT)	616	68.99%
	Transfemoral (TF)	219	24.53%
	Foot amputations	31	3.47%
	Other Amputations	27	3.02%
Cause of Amputation	Diabetes Mellitus (DM)	499	55.88%
	Cancer	165	18.48%
	Trauma	104	11.65%
	Congenital	57	6.38%
	Peripheral Vascular Disease (PVD)	35	3.92%
	Other Causes	33	3.68%
Age Group	Children (0–16)	66	7.39%
	Young Adults (17–30)	136	15.23%
	Middle-Aged (31–45)	179	20.04%
	Older Adults (45+)	512	57.33%
Gender	Male	692	77.50%
	Female	201	22.50%

Regarding the level of amputation, TT amputations were the most frequently observed anatomical level among participants (Table 1). The TT to TF amputation ratio was 2.8:1, underscoring the prevalence of TT amputations in this population. TF amputations accounted for 219 cases (24.53%), while foot amputations (31 cases, 3.47%) and other amputation types (27 cases, 3.02%) were much less frequent.

The demographic analysis of lower extremity amputations (LEA) in this study reveals notable age and gender trends. The most affected age group were individuals over 45 years, accounting for 512 patients (57.33%), with a mean age of (48.43, SD = 20.42 years). Middle-aged adults (31–45 years) represented 179 cases (20.04%), followed by young adults (17–30 years) with 136 cases (15.23%). The lowest proportion was found in children (0–16 years), with 66 cases (7.39%). A significant gender disparity was observed in LEA incidence, with males comprising 692 cases (77.50%), nearly 3.5 times the number of female cases (201 cases, 22.50%).

The findings indicate that diabetes mellitus (DM) is the leading cause of amputation, accounting for 55.88% of cases. Cancer and trauma follow, contributing (165 cases, 18.48%) and (104 cases, 11.65%) respectively. Congenital conditions accounted for 57 cases (6.39%) of amputations, while peripheral vascular disease (PVD) was responsible for 35 cases (3.92%). The least common category, other causes, contributed to 33 cases (3.68%).

**Fig 1:**
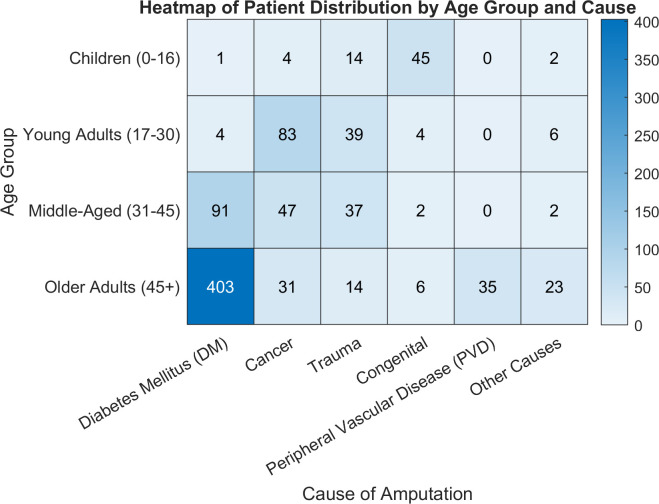
The heatmap representation of the distribution of patients by age group and cause of amputation reveals distinct patterns across different age groups and conditions.

Among children (0–16 years), the majority of cases are attributed to congenital causes (45), followed by trauma (14) and cancer (4), with a small proportion (2) associated with other causes. (Fig 1). In young adults (17–30 years), cancer emerges as the dominant cause of amputation (83 cases), followed by trauma (39), while congenital and peripheral vascular disease cases are notably less frequent. Middle-aged adults (31–45 years) show a more balanced distribution, with trauma (37) and DM (91) as the primary causes, while congenital causes remain minimal (2). For older adults (45 + years), the most frequent causes are DM (403 cases) and PVD (35), with trauma (14) and cancer (31). The heatmap (Fig 1) clearly highlights the rising prevalence of diabetes and PVD with increasing age, as well as the significant impact of congenital causes in children and cancer in young adults.

The correlation analysis between age group and cause of amputation revealed a significant positive relationship (r = 0.677, p < 0.01), indicating a strong association between age and the cause of amputation. This suggests that certain amputation causes are more prevalent within specific age groups, potentially guiding targeted prevention and intervention strategies.

The ANOVA results also showed significant differences in age across different causes of amputation (F = 242.23, p < 0.001). Further examination of mean ages by cause indicates that the average age for patients with DM as a cause was 58.53 years (SD = 13.12), significantly higher than for other causes.

### Discussion

This retrospective study examined the demographic characteristics of individuals with lower extremity amputations (LEA) in Jordan from 2017 to 2023. The final study population was 893 patients. This sample size encompasses diverse demographic and clinical characteristics and reflects the population of individuals with amputations across the country. The findings can therefore be considered representative of national trends, acknowledging that complete population data was not feasible to obtain.

Key findings indicate that transtibial (below-knee) amputations were predominant, with a transtibial-to-transfemoral (TTA to TFA) ratio of 2.8:1. The gender distribution showed a male-to-female ratio of 3.5:1, with a mean age of 48.43 (SD = 20.42) years. Diabetes mellitus (DM) emerged as the leading cause of LEA, highlighting its significant impact on lower limb health in Jordan. Additionally, 8% of individuals experienced bilateral LEA, with DM as the primary cause. (Table 1, Fig 1).

The predominance of TT amputations TF amputations is a widely observed pattern globally and is consistent with findings from previous studies in Jordan [7]. However, the higher TT to TF ratio (2.8:1) reported here (Table 1) exceeds that typically observed in developed countries, such as in the US it was found to be 1.29:1 [13]. A prior study conducted in Jordan [14] attributed this disparity to insufficient early assessments and delayed referrals, which was stated to often lead to adverse events that ultimately preclude effective limb-saving interventions. Such delays may increase the likelihood of disease progression, limiting the feasibility of preserving the affected limb. Supporting this, the EuroDIALE study found that only 27% of patients with diabetic foot ulcers (DFU) persisting for over three months were referred for specialized care, underscoring the impact of referral delays on amputation risk [15].

The observed male-to-female ratio (3.5:1) in the prevalence of LEA in Jordan is consistent with previous findings in Jordan [6] and as in the Middle East and other developing countries [16, 17]. This disparity may be partially attributed to the higher incidence of DM and PVD among males compared to females. Additionally, the progression of these underlying conditions tends to be more severe in males, particularly at older ages, leading to a higher likelihood of requiring major LEAs as a treatment option [1]. These factors underscore the need for targeted prevention strategies that address the gender-specific risks associated with these conditions.

The mean age for LEA presented in this study was (48.1) years which aligns with findings from several studies in developing countries. A study by Al-Worikat et al. (2003) in southern Jordan reported a mean age of 46 years, while a study conducted in Saudi Arabia by [18] found a mean age of 43 years. In contrast, the mean age for LEA in developed countries tends to be in the 70s [19]. This disparity can be largely attributed to the high prevalence of diabetes mellitus (DM) and its associated complications in our patient population. DM, especially when acquired at a younger age, significantly increases the risk of amputation at an earlier age [20]. These findings emphasize the urgent need for improved management and prevention strategies for DM to reduce the incidence of early-onset amputations.

As highlighted in the results section, DM was identified as the leading cause LEA in Jordan, with its related complications contributing significantly to the high incidence of amputation. This finding aligns with studies conducted in other developing countries, such as Bahrain and the Caribbean, where DM is also reported as the primary factor leading to LEA [2, 21]. Conversely, in countries like Nigeria and Iran, trauma has been identified as the leading cause of LEA [22, 23]. In contrast, developed countries report peripheral vascular disease (PVD) and DM as the primary causes of amputation [24].

This present study further revealed a significant increase in the incidence of LEA with age, with individuals older than 45 years exhibiting the highest rates of amputation (Table 1). Among this age group, DM and its complications were the predominant causes of amputation (Table 1, Fig 1). This trend is consistent with the rising prevalence of DM in Jordan, which increased from 13% in 1994 to 23.7% in 2017 [25].

Diabetic individuals are at a considerably higher risk of amputation compared to non-diabetic individuals [26]. Diabetes-related foot infections, particularly diabetic foot ulcers (DFU), have been identified as major contributors to LEA in developing countries [27].

In Jordan, the incidence of DFU is reported to be 4.6%, which is notably higher than the global average of 2% [28]. It is estimated that one in three individuals with DFU will undergo amputation [29]. The severity of DM and its complications generally increases with age, making it a leading cause of major LEA in individuals over the age of 45 [14]. These findings are in agreement with studies conducted in other developing nations, including India and Nigeria [17, 20].

Cancer is the second most common cause of LEA, with a percentage of 18.48% (Table 1, Fig 1). This proportion is notably higher than that reported in previous studies on the Jordanian population and other regional populations [6, 21]. This finding is attributed to the fact that bone tumors are among the top five cancers affecting children below 14 years old in Jordan, as reported by the Jordan Cancer Registry in 2018 and 2019 (Jordan Cancer Registry, 2018, 2019). They also reported that TF amputation was the most common level of amputation among cancer patients. This finding aligns with existing literature, which indicates that 80–90% of bone tumors occur in the metaphysis of long bones, often in the proximal tibia and distal femur, leading to a higher frequency of TF amputations [30]. The young adults and children are the most affected age groups, a finding that corroborates previous studies [30]. Future research is needed to explore the underlying reasons for the increased incidence of amputations in cancer patients and to identify potential strategies to reduce this number.

The ANOVA results revealed significant age differences among the causes of amputation with DM-related amputations affecting older adults. This variation highlights the need for age-specific preventative strategies, such as DM management in older adults and trauma prevention for middle-aged individuals.

This study emphasizes the need to increase awareness and preventive measures for diabetic foot complications, a leading cause of amputations. Educating healthcare professionals, patients, and the public on risk factors and preventive care for diabetic foot complications can be achieved through targeted public health campaigns, healthcare provider training, and comprehensive patient education materials. These materials could focus on topics such as regular foot inspection, nail and skin care, appropriate footwear selection, and recognizing when to seek medical attention, which empowers patients to self-manage their health effectively. Additionally, cancer-related amputations warrant further investigation, as understanding these cases more deeply could lead to improved treatment protocols that may reduce their incidence. Encouraging research that aims to develop multidisciplinary care approaches and early intervention techniques for cancer patients could have a significant impact on reducing the need for amputations.

Policymakers are encouraged to integrate these strategies into healthcare frameworks to improve patient outcomes. Establishing regular diabetic foot screenings as part of standard care protocols could be particularly impactful. By prioritizing these preventive measures and healthcare provider training, policymakers can help decrease the prevalence of amputations and lessen the resulting functional impairments.

A major strength of this study is its comprehensive dataset, which spans six years and includes nearly 900 patients, making it one of the most extensive analyses of LEA demographics in Jordan. Furthermore, the study provides comparative insights with regional and international trends, allowing for meaningful interpretation within a global context.

One limitation of this study is the exclusion of 176 records due to missing data in key variables such as age, gender, cause, or level of amputation. Although necessary to ensure the validity of the analysis, these exclusions may introduce selection bias. However, the missingness appeared to be random and primarily administrative in nature such as incomplete file transfers or issues with handwritten documentation. This is unlikely to be systematically associated with patient characteristics or outcomes. Additionally, the reliance on paper-based medical records may have introduced further inconsistencies, including illegible handwriting, documentation errors, and variability in record-keeping practices across healthcare providers. The absence of a standardized data format also limited data retrieval efficiency and overall reliability. Future studies are encouraged to implement electronic health records (EHRs) or similar digital data systems, which can improve documentation consistency, reduce missing data, and enable more accurate, longitudinal analyses of patient outcomes.

## Conclusion

This study concludes that TTA is the most prevalent amputation level, with individuals over the age of 45 being most affected and a male-to-female amputation ratio of 3:1. The study’s findings underscore DM as the leading cause of lower limb amputations in Jordan. This research not only highlights the demographic and clinical characteristics of amputation cases but also calls for the development of proactive health policies focused on foot health and early intervention for diabetes-related complications.

The study is intended to serve as a catalyst initiative aimed at enhancing patient outcomes and encourages healthcare policymakers to support healthcare professionals in promoting national foot health awareness, thereby reducing the incidence of LEAs.

## Supporting information

S1 Table Summary of missing values for each variable in the dataset. (DOCX)

S2 Checklist STROBE checklist for cross-sectional studies. (DOC)

S3 File Inclusivity in global research questionnaire. (DOCX)
