# Supplementary material for: Health implications of lower extremity amputations in Jordan: A retrospective analysis of demographic patterns and causes
Source: PLoS One. 2025 Jul 24;20(7):e0329149. doi: 10.1371/journal.pone.0329149 (PMC12289060; doi:10.1371/journal.pone.0329149)
Supplement: S1 Table — (DOCX) [file pone.0329149.s001.docx]

| **Variable** | **Number of records missing** | **Percentage of total records (%)** |
| --- | --- | --- |
| Age | 58 | 5.43% |
| Gender | 51 | 4.77% |
| Cause of amputation | 44 | 4.12% |
| Amputation level | 32 | 2.99% |

**Table S1.** Summary of missing data by variable (N = 1,069)

This table shows the number and percentage of records missing each key variable prior to exclusion. Some records were missing more than one variable; therefore, the total number of missing entries across variables (185) is greater than the number of unique records excluded (176).
